# Electronic Properties of Parallel-Aligned Arrays of Carbon Nanotubes

**DOI:** 10.3390/ma18174095

**Published:** 2025-09-01

**Authors:** Bartosz Brzostowski, Jacek Wojtkiewicz

**Affiliations:** 1 Faculty of Physics and Astronomy, University of Wrocław, pl. M. Borna 9, 50-204 Wrocław, Poland; bartosz.brzostowski@uwr.edu.pl; 2 Faculty of Physics, Warsaw University, Pasteura 5, 02-093 Warszawa, Poland

**Keywords:** carbon nanotubes, carbon allotropes, density functional theory, band structure, energy gap

## Abstract

We present results of electronic properties’ DFT calculations, i.e., band structure, density of states and energy gap for arrays of parallel-ordered infinitely long small-diameter carbon nanotubes (CNTs). It turns out that if the distance between CNTs is sufficiently large (of the order 7.5 Å or greater), the electronic properties of arrays are very close to isolated CNTs. However, when CNTs in arrays are forced to be at a distance of the order of 5 Å, they undergo radical structural rearrangements, leading to completely different structures: graphite-like or three-dimensional lattice intermediates between graphite and diamond. Electronic properties are also rebuilt in a drastic manner.

## 1. Introduction

Carbon nanotubes (CNTs)—quasi-one-dimensional allotropes of carbon, being cylindrical tubes with nanometers in diameter and up to several millimeters in length—have attracted significant research interest ever since their discovery due to their outstanding properties: electronic, optic, mechanical and superconductive [1,2]. From the authors’ personal point of view, their electronic, optic and excitonic properties are particularly interesting, due to the possible applications of CNTs as active layers in solar cells [3,4]. Electronic properties of isolated CNTs have been the subject of numerous theoretical studies (see, for example, [4,5,6]).

However, CNTs in experiments rarely appear as independent entities; most often, they form random arrays of disordered fibers, or are placed into “matrices” of external host.

At present, the structures of parallel-aligned nanotubes (carbon ones, but also built from another elements, such as titanium, boron, etc.) are attracting increasing attention [7]. Their potential applications serve, among others, as adhesive films, blackbody absorbers, biological sensors, gas sensors, and electron-emitting materials [8,9,10,11,12,13,14,15].

From this point of view, theoretical studies on arrays of parallel-oriented CNTs are highly desirable. In our paper, we present results for electronic properties of arrays of parallel-aligned CNTs.

We study the band structure, densities of states (DOSs) and, in particular, the main energy gap. Our overall goal is to carry out a systematic study of many CNTs differing in diameter and chirality on a broad range. In the present paper, we begin such a study by examining small-diameter objects and report results for some CNTs (*n* = 2 and 3). The general conclusion is that the electronic structure of arrays does not differ significantly from the properties of isolated ones for distances between CNTs that are sufficiently large. However, we observe that when they are sufficiently close to each other, they undergo a structural metamorphosis to a graphite-like state [16], or even more complicated structures. This is accompanied by radical structural rebuilding.

These results suggests that arrays of small-diameter CNTs are not stable and that stability can be expected only for arrays of larger-diameter CNTs [13].

## 2. Computational Methods

We undertook a systematic exploration of small-diameter carbon nanotubes to examine their electronic properties. We performed first-principle calculations within the framework of the density-functional theory (DFT) as implemented in SIESTA 4.1.5 [17,18].

For carbon atoms, we used norm-conserving pseudo-potential generated with the Troullier–Martins scheme [19,20] for core radii equal to 1.58 Bohr for all valence orbitals. The exchange and correlation effects were accounted for by generalized gradient approximation (GGA) with PBE exchange–correlation potential [21]. The ideal atomic positions in cells with a fixed distance of 1.41 Å between C atoms were generated by the TubeASP applet [22]. The optimized atomic structures were obtained by fully relaxing both atomic positions as well as cell parameters by using the conjugate gradient (CG) method. The optimization algorithm varied the atomic structure of the system until all forces were smaller than 10^−6^ eV/Å. All parameters critical for convergence, such as the k-point mesh and the energy cutoff, were carefully tested to ensure the most accurate results. As part of the tests, mesh cutoff from 1000 to 2000 Ry was checked. For the study of electronic properties, it turned out that the value of 1000 Ry is large enough.

The unit cells were constructed so that the axis of the nanotube was oriented in the *z* direction; in the *xy* plane, the nanotubes were distributed on a triangular lattice by imposing periodic boundary conditions. The nanotube axis was located in the center of the *xy* plane of the unit cell, and the cell vectors defined the distance between the nanotubes. We initially checked for larger values of the distance between the nanotubes (30, 25 and 20 Å, respectively), and it turned out that even for 15 Å the electron properties of the nanotubes remain unchanged.

Since unit cells were used for calculating the properties of isolated nanotubes, which are small and contain few atoms (in fact, the size of the unit cell in the *z*-direction is of the order of several Å) for some nanotubes, it was necessary to use larger values of parameters such as plane-wave cutoff (mesh cutoff) and Brillouin zone sampling (*k*-grid Monkhorst–Pack).

Tests have shown that the 1 × 1 × *M* Monkhorst–Pack grid [23] is good enough for coordinates *x*, *y* and that only the *M* (in *z*-th direction) value is significant for convergence. For the smallest unit cells, the value of this parameter was tested up to 3000. To investigate electronic properties, such as the main energy gap, a sufficiently good value of this parameter is *M* = 1200. For nanotubes with a clearly non-zero energy gap, a convergence is obtained for smaller values of the *M* parameter. On the other hand, nanotubes with a gap of zero or close to zero make convergence difficult.

Moreover, let us comment on the modification of the computational methodology when the CNT distance is decreasing. When the inter-tube distance is large enough (7.5 Å or more), the volume size of an elementary cell is large, so the *k*-grid can be still taken as 1 × 1 × *M* (nanotubes are still clearly separated from each other and the reciprocal lattice vectors are relatively small due to the size of the real lattice vectors). However, when CNTs are closer and closer to each other, the size of an elementary cell of an array decreases, and the structure resembles a true three-dimesional one, where the interatomic distance is comparable in all directions. In such a case, one should take a *k*-grid of the form K×K×M, where *K* should be appropriately determined. In our calculations, the value K=50 was sufficient to achieve convergence in the case where nanotubes located close to each other retained their structure. However, for small CNT distances (about 5 Å or less), we encounter another difficulty: it is difficult to achieve the convergence. As a consequence, calculations with a more subtle *k*-grid (i.e., larger values of *K*) become massively time-consuming. In addition, we have to work with radical changes in structure, and a large number of steps of molecular dynamics is necessary. This aspect will be the subject of further research; in this work, we were interested in how close the nanotubes can be to each other and how this affects their electronic properties.

Stability of CNTs: In our previous papers [3,5], we examined the stability of isolated tubes via a calculation of phonon spectra (with the finding being that if all eigenfrequencies are positive, then the configuration is stable). However, such calculations are more time-consuming than the calculations of electron properties. Here, we have only partial results, and we do not present them in this paper. On the other hand, our previous calculations [3,5] of phonon frequencies for single CNTs tell us that all of them are stable. We extrapolate these results for arrays of CNTs, but we realize that in order to be sure, additional calculations would be desired—which are not possible for us at present, as they are very demanding from a computational point of view.

## 3. Results

### 3.1. Isolated CNTs

**Figure 1:**
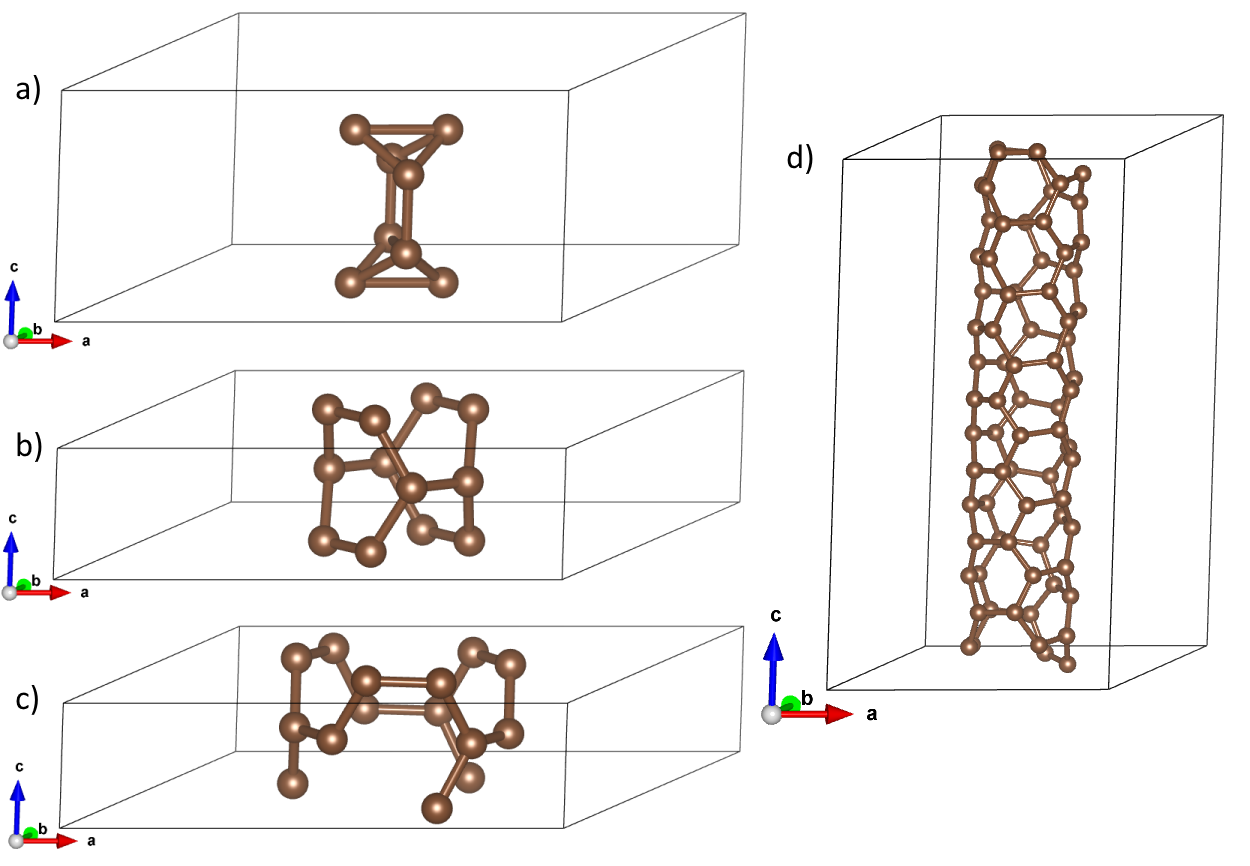
Elementary cells for optimized structure for (**a**) (2,0), (**b**) (2,2), (**c**) (3,3), and (**d**) (3,2) carbon nanotubes, respectively.

CNTs are characterized by two parameters, (n,m), where *n* is a natural number greater than 1 (*n* is roughly related to a diameter), whereas *m* denotes the chirality parameter, which—for a given *n*—takes values from 0 to *n* [1]. In our study, we compute properties of small-diameter CNTs: *n* take values 2 and 3. We investigated two conducting (i.e., zero-gap) and two insulating (non-zero gap) CNTs: Diameter-2 series: (2,0) (non-zero gap) and (2,2) (zero gap). Diameter-3 series: (3,2) (non-zero gap) and (3,3) (zero gap). Their structures are displayed in Figure 1 for elementary cells.

**Figure 2:**
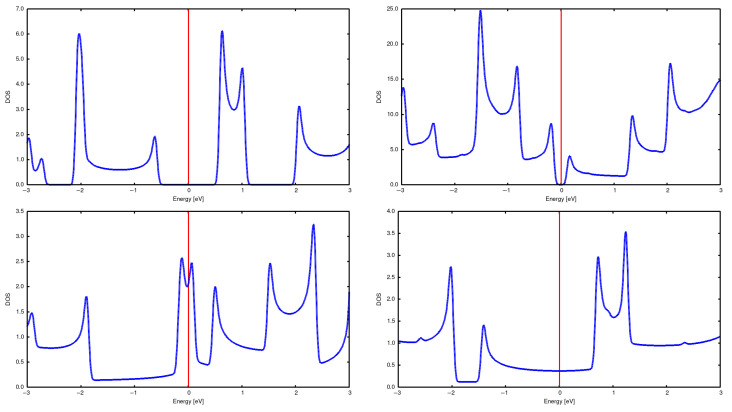
DOSs for single CNTs: (2,0) (**top left**), (2,2) (**bottom left**), (3,2) (**top right**), and (3,3) (**bottom right**).

**Figure 3:**
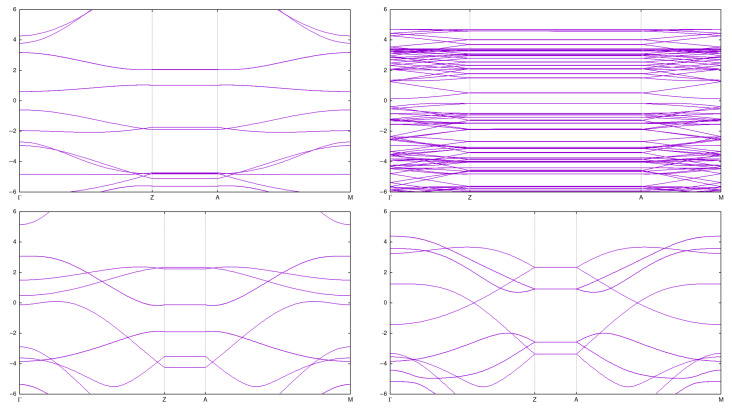
Band structure for single CNTs: (2,0) (**top left**), (2,2) (**bottom left**), (3,2) (**top right**), and (3,3) (**bottom right**).

Properties of these CNTs have were calculated [5,6], but we reproduce them here for the sake of completeness and for a comparison with the results for arrays. See Figure 2 and Figure 3, as well as Table 1.

### 3.2. CNT Arrays

**Figure 4:**
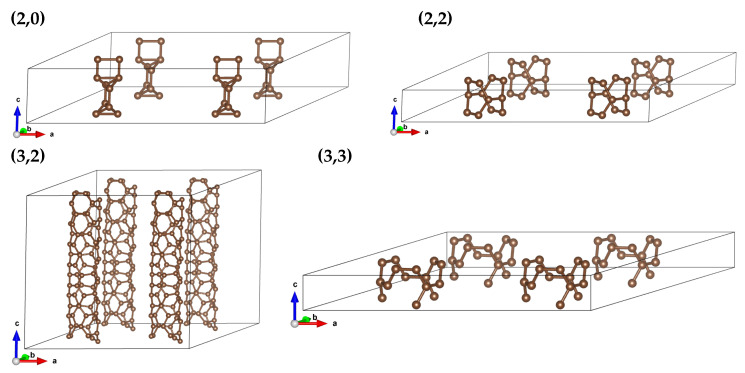
Structure of arrays for (2,0), (2,2), (3,2), and (3,3) CNTs. Only elementary cells of CNTs are presented.

We repeated calculations for arrays of parallel-oriented CNTs. We considered nanotube systems distributed over a 2D triangular lattice (to achieve the ‘close packing’) with nanotube distances of 15.0, 10.0, 7.5, and 5.0 Å. ‘Distance’ represents the distance between the middle points of CNTs. The elementary cell possess angles 60, 90 and 90 deg, so the notation of special points of the Brillouin zone can be seen as not standard. The array structure is illustrated in Figure 4 for elementary cells of every CNT.

We have performed calculations for elementary cells containing one, four and nine CNTs. The corresponding notations are 1 × 1, 2 × 2 and 3 × 3. This means that an elementary cell is multiplied in directions perpendicular to the CNT axis. The goal of this methodology is simple: the larger the elementary cell, the better the precision of calculations.

In all presented DOS plots and band structures, the Fermi level shifted to 0.

Results for band structures and DOSs are very similar for single (isolated) CNTs and their arrays for inter-tube distances of 15 Å and 10 Å. When the distance is equal to 7.5 Å one observes some changes, but they are not significant. See Table 2. The geometric structures of CNTs in arrays are also similar to those for the single tube. However, the situation changes dramatically when the distance is forced to be 5 Å for all the three quantities: DOSs, band structures and gaps, and the geometry of the crystal lattice. We describe these changes in more details for every individual CNT.

- (**2,0**) CNT

The band structure changes radically for distances less than 5 Å. The structures of DOSs and bands change completely and the energy gaps close, so the system becomes a conductor (for the array of 2 × 2 CNTs).

**Figure 5:**
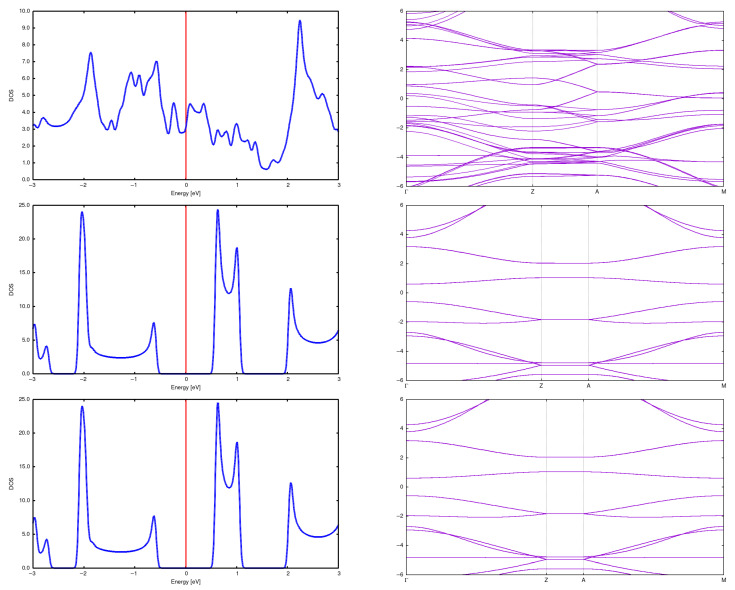
DOS and band structure for a (2,0) CNT in a 2 × 2 array with *d* = 5 Å (**top**), *d* = 7.5 Å (**center**) and *d* = 10 Å (**bottom**). For a distance equal to 5 Å one still observes the nanotube structure, but it is significantly deformed in comparison to the isolated case. Moreover, the energy gap closes.

**Figure 6:**
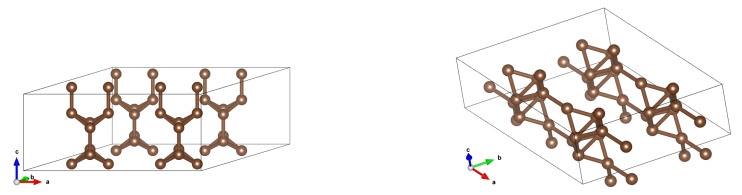
Structures after optimization for (2,0) CNTs in 2 × 2 arrays for inter-tube distances 5 Å (**left**) and 4 Å (**right**). It is seen that for 5 Å the ‘tube-like’ structure is preserved, whereas for 4 Å a completely new structure appears— planes that resemble nanotubes. Only elementary cells of a (2,0) CNT and a reorganized one are shown.

**Figure 7:**
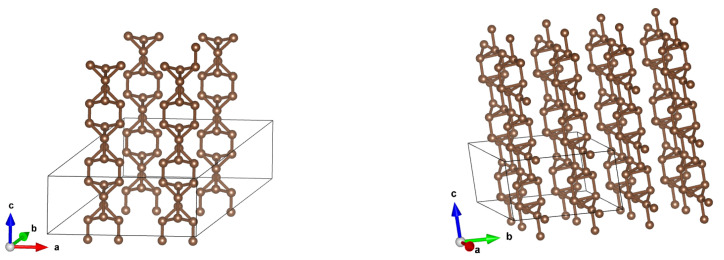
Structures after optimization for (2,0) CNTs in 2 × 2 arrays for inter-tube distances 5 Å (**left**) and 4 Å (**right**) in a more illustrative manner.

See Figure 5. It is accompanied by a complete reorganization of a crystal structure to graphene-like one. See Figure 6, and in a more illustrative manner, see Figure 7.

For this nanotube, the results presented in Table 2 for an inter-nanotube distance of 5 Å may be viewed as confusing. We have verified in several independent calculations that the gap closes for the 2 × 2 case with four nanotubes in the supercell; for the 3 × 3 case (9 nanotubes in the cell), the main energy gap is almost three times smaller than for an isolated nanotube. Moreover, the result for one nanotube in the unit cell (1 × 1) indicates a strong (self) interaction between nanotubes from adjacent unit cells. In this case, the HOMO-LUMO gap increases by 30 percent compared to the value of 1.2 eV observed for larger distances between nanotubes. For the 2 × 2 system, the nanotubes are significantly distorted compared to the isolated nanotube; in the 3 × 3 case, the changes are not as pronounced. Changes in the structure, electronic properties and the fact that the nanotubes clearly interact with each other suggest that in order to clearly determine whether, at such a small distance between the nanotubes, they still have metallic characteristics, one should consider a larger structure with a larger number of nanotubes in the calculation cell and/or include such structure in the calculations interactions, which would better describe the system under consideration. In this aspect, our study of (2,0) CNT arrays is not complete; nonetheless, structural rearrangement of the array is viewed.

- (**2,2**) CNT

**Figure 8:**
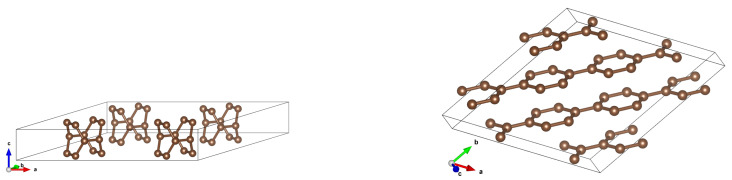
A (2,2) CNT with optimized structures for initial distances 7.5 Å (**left**) and 5 Å (**right**). It is seen that for 5 Å a graphene-like structure emerges.

**Figure 9:**
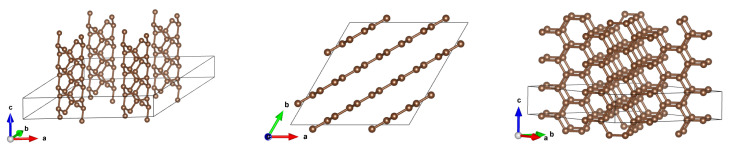
A (2,2) CNT with optimized structures for initial distances 7.5 Å (**left**) and 5 Å (**middle** and **right**) presented in a more illustrative manner.

**Figure 10:**
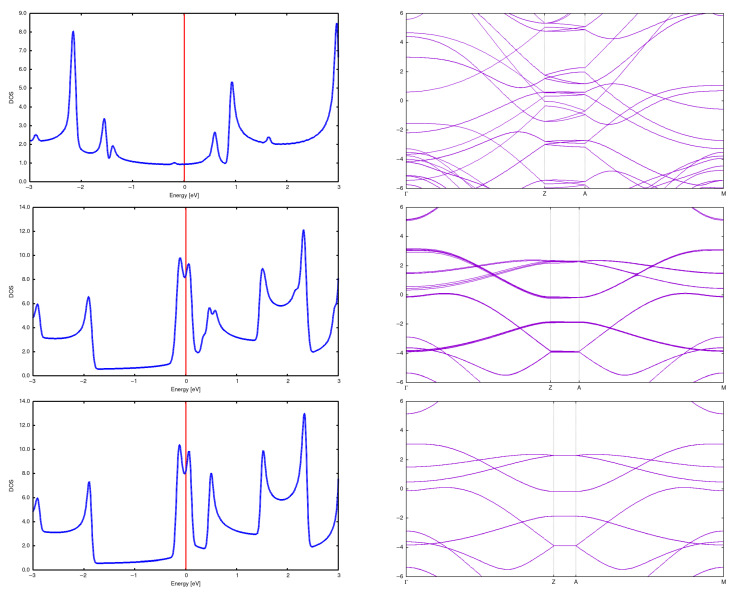
DOS and band structure for a (2,2) CNT in a 2 × 2 array with *d* = 5 Å (**top**), *d* = 7.5 Å (**center**) and *d* = 10 Å (**bottom**).

In the case of (2,2) CNTs, the structure rebuilds at a distance of about 5 Å; the result is a set of graphene-like planes (‘strange graphite’). See Figure 8 and Figure 9. The bands and DOSs also undergo a dramatic metamorphosis, but the system is still gapless. See Figure 10.

- (**3,2**) CNT

In this case, we have no definite results as the optimization process does not converge even after a large number of steps. Perhaps the optimized structure is complicated and includes a large number of atoms in an elementary cell.

- (**3,3**) CNT

**Figure 11:**
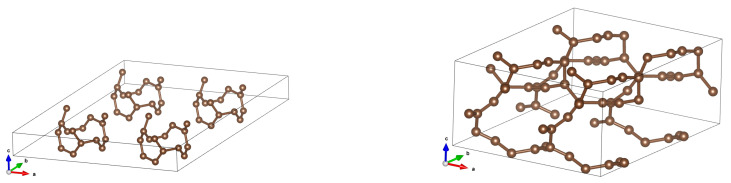
A (3,3) CNT with optimized structures for initial distances 7.5 Å and 5 Å. It is seen that something intermediate between a diamond and graphite appears.

**Figure 12:**
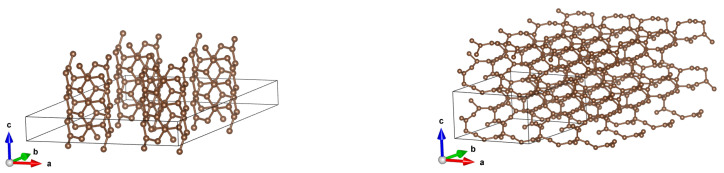
A (3,3) CNT with optimized structures for initial distances 7.5 Å and 5 Å–the same as in Figure 11, but with larger fragments of structures being shown.

**Figure 13:**
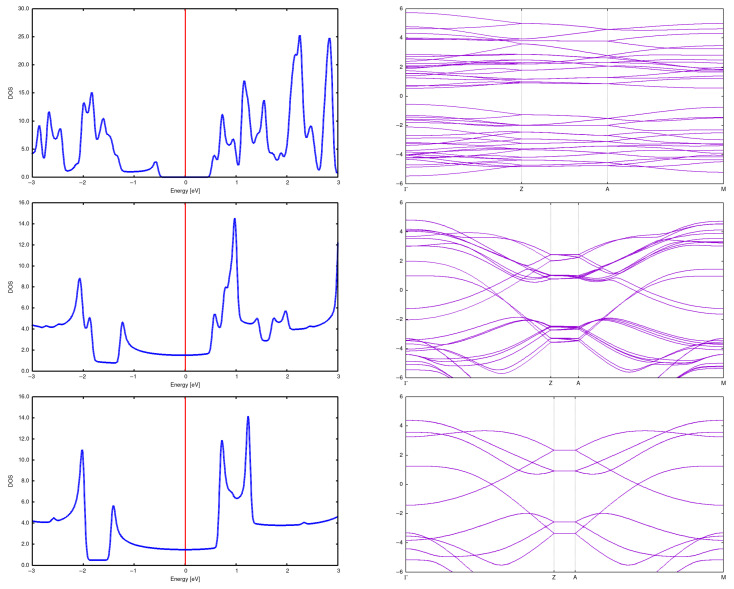
DOS and band structure for a (3,3) CNT in a (3 × 3) array with *d* = 5 Å (**top**), *d* = 7.5 Å (**center**) and *d* = 10 Å (**bottom**). It can be seen that the DOS and band change dramatically for a distance 5Å; in particular, an energy gap opens.

Here, we present structures for initial distances 7.5 Å and 5 Å. It is seen that the structure changes completely, resulting in something intermediate between a diamond and graphite. See Figure 11 and Figure 12 for a more illustrative representation. Notably, DOSs and bands rebuild in a profound manner. See Figure 13.

## 4. Summary, Perspectives of Further Investigations

We have calculated the electronic properties of arrays of small-diameter carbon nanotubes. As far as we know, existing experimental results concern only (small-diameter) CNTs in matrices—we do not know results for isolated CNTs or their arrays.

It turned out that for large inter-tube distances, the DOS and band structures do not differ from the case of isolated CNTs. However, for smaller distances, the optimized structure differs radically from initial ones, which also implies a radical change in the DOS and band structures. Structures resulting after metamorphoses are quite diverse: graphite-like (emerging from a (2,2) CNT array), array of ‘strange CNTs’ (emerging from a (2,0) CNT array), and something between a diamond and graphite (emerging from a (3,3) CNT array; in this case, it resembles carbon fluorides reported in [24] to some extent). The result of our study can be viewed as a suggestion pointing to small-diameter CNTs (of series 2 and 3) being thermodynamically unstable.

It would be beneficial to continue these calculations for the following reasons:

- Make further tests, especially including functionals, providing a good description of van der Waals forces.
- Determine the equilibrium distance between CNTs (if it exists). On the ground of the present calculation, we conjecture that such arrays does not exist for small-diameter CNTs and are stable only for larger-diameter CNTs. This last case has been examined, among others, in [13], where it has been established that large-diameter CNTs preserve their structures under decreasing inter-tube distances, only undergoing deformations. One can guess that in this case, the distance between the walls of CNTs is the order of 3.5Å, like the distance between graphene layers in graphite. We hope to address this problem in the future; the approach of [13] is a combination of classical molecular dynamics (MD) simulations and continuum analyzis, and it has been performed for large-diameter CNTs (*n* = 17 or more). We hope that this DFT study can shed additional light on the behavior of CNT arrays for *n* values ranging from 3 to approximately 15.
- For equilibrium-distance arrays, the following properties are of particular interest: the geometry of CNTs; DOS and band structures; optical properties; and exciton diffusion in arrays. All of these properties are crucial for the concept of CNT-array-based solar cells. Unfortunately, their computational demand seems to be enormous.
- It would be beneficial for us to extensively examine the carbon allotropes emerging from small-diameter CNTs upon decreasing the inter-tube distance, such as compute radial distribution functions, coordination analyzis, electronic localization function (ELF) plots, and thermodynamic functions.

## Figures and Tables

**Table 1:** HOMO-LUMO gaps [eV] for isolated nanotubes.

CNT	(2,0)	(2,2)	(3,2)	(3,3)
H-L gap	1.20	0.00	0.29	0.00

**Table 2:** HOMO-LUMO gap.

d [Å]	(2,0)			(2,2)			(3,3)		(3,2)	
	1 × 1	2 × 2	3 × 3	1 × 1	2 × 2	3 × 3	1 × 1	2 × 2	1 × 1	2 × 2
5.0	1.58	0.00	0.445	0.01	0.01	0.00	2.15	1.10	–	–
7.5	1.20	1.2	1.20	0.00	0.00	0.00	0.02	0.00	0.55	–
10.0	1.20	1.20	1.20	0.00	0.01	0.01	0.00	0.00	0.29	0.29
15.0	1.20	1.20	1.20	0.00	0.01	0.01	0.00	0.00	0.29	0.29

## Data Availability

The authors declare that the data supporting the findings of this study are available within the paper.
